# Interpretation of solution scattering data from lipid nanodiscs

**DOI:** 10.1107/S1600576717018441

**Published:** 2018-02-01

**Authors:** Vito Graziano, Lisa Miller, Lin Yang

**Affiliations:** a Brookhaven National Laboratory, PO Box 5000, Upton, NY 11973-5000, USA

**Keywords:** lipid nanodiscs, solution scattering, small-angle X-ray scattering, wide-angle X-ray scattering, SAXS/WAXS, modelling

## Abstract

The structural information contained in solution scattering data from empty lipid nanodiscs is examined in the context of a multi-component geometric model. X-ray scattering data were collected on nanodiscs of different compositions at scattering vector magnitudes up to 2.0 Å^−1^. Through the calculation of the partial form factor for each of the nanodisc components before the isotropic average, structural parameters in the model were correlated to the features observed in the X-ray scattering data and to the corresponding distance distribution function. It is shown that, in general, the features at ∼0.3–0.6 Å^−1^ in the scattering data correlate to the bilayer structure. The data also support the argument that the elliptical shape of nanodiscs found in model fitting is physical, rather than an artefact due to the nanodisc size distribution. The lipid chain packing peak at ∼1.5 Å^−1^ is visible in the data and reflects the lipid bilayer phase transition. The shape change in the distance distribution function across the phase transition suggests that the nanodiscs are more circular in the fluid phase. The implication of these findings for model fitting of empty and protein-loaded nanodiscs is discussed.

## Abstract 2

Solution scattering data from lipid nanodiscs are interpreted on the basis of X-ray scattering data in an extended range of scattering vectors.

## 1. Introduction   

Structural studies of membrane proteins are challenging because biophysical and biochemical characterization tools generally require homogenous solution samples. In order to avoid aggregation, one must first solubilize the membrane protein by concealing its hydrophobic surface using amphiphilic molecules. The resulting membrane protein–amphiphile complexes are then studied. While detergents have traditionally been used as the solubilizing agent, they can affect the activity of the protein (see *e.g.* the review by Seddon *et al.*, 2004 ▸). Detergents can also complicate the accurate measurement of small-angle X-ray scattering (SAXS) data from solubilized proteins, where the background scattering from the buffer and free detergent micelles must be subtracted before data analysis can proceed. Whether this subtraction is even possible in a three-component system composed of protein–detergent complexes, free micelles and aqueous buffer depends strongly upon the type of detergent (*e.g.* as discussed on the web site of the SIBYLS beamline at the Advanced Light Source, Berkeley, California, USA; https://bl1231.als.lbl.gov/). Furthermore, detergent micelles in a protein–detergent complex exhibit a different average electron density from the protein itself, with unknown structure, adding to the complexity of the data analysis. As a result of these fundamental limitations, there have been active efforts seeking other amphiphiles that are more suitable for structural studies. These alternatives, including amphipols (Tribet *et al.*, 1996 ▸), bicelles (Sanders & Landis, 1995 ▸) and lipid nanodiscs, have been successful to varying degrees. The purpose of this study is to critically evaluate the potential of using solution scattering to determine the low-resolution structures of membrane proteins embedded in lipid nanodiscs.

Compared with other amphiphiles, lipid nanodiscs have distinct advantages. Each nanodisc consists of a planar lipid bilayer surrounded by two membrane scaffold proteins (MSPs) (Bayburt *et al.*, 2002 ▸). The MSPs cover the hydrophobic surface of the lipid bilayer and define the size of the lipid nanodisc. The composition of the lipids in the bilayer can be precisely controlled to mimic the environment required for the membrane protein to function. Finally, the size of the nanodisc can be varied by selecting the appropriate MSP sequence length to accommodate the membrane protein that is to be incorporated into the nanodisc.

So far, lipid nanodiscs have found wide application in various structural characterization methods, including NMR and cryo-electron microscopy (cryo-EM) (Bayburt & Sligar, 2010 ▸; Denisov & Sligar, 2016 ▸). There is also strong interest from the solution X-ray and neutron scattering community to make use of nanodiscs. Since the length of the MSP defines the size of the nanodisc, membran-protein-embedded nanodiscs are expected to meet the requirement of high particle monodispersity for solution scattering. Furthermore, background scattering from free lipid molecules in the solution is negligible, owing to the very low critical micelle concentration of lipids. Therefore high-quality solution scattering data can be readily obtained.

For many in the solution scattering community, nanodiscs bear the high hope of solving the shape modelling problem for membrane proteins. These modelling methods are sophisticated fitting routines that optimize the calculated scattering intensity based on an evolving low-resolution structural model against the experimentally measured scattering data. These methods have been highly successful for soluble proteins, which can be treated as shape envelopes within which the scattering density is uniform. In contrast, particles that consist of components of different densities, such as protein–detergent complexes or protein–DNA complexes, present too many variables for the modelling algorithm to converge reliably and so additional inputs must be included. For instance, contrast variation and matching using neutron scattering can be used to provide additional constraints to the model (Skar-Gislinge & Arleth, 2011 ▸). Since the size of the nanodisc is well defined, the structure of the empty nanodisc, *i.e.* one without membrane protein, can be characterized first. This known structure can then be used as an input in the modelling process for the membrane protein structure, which can be assumed to be uniform in scattering density. This approach has been demonstrated recently by Skar-Gislinge *et al.* (2015 ▸).

In this report, we focus on the structural characterization of empty nanodiscs, which were prepared following well established protocols. We present data measured from several nanodisc constructs at *q* up to 0.6 Å^−1^, as well as from 1,2-dimyristoyl-*sn*-glycero-3-phos­pho­choline (DMPC) nanodiscs at *q* up to 2.0 Å^−1^ and above and below the DMPC phase transition temperature. In our discussion, we make use of the software tool *WillItFit* (Pedersen *et al.*, 2013 ▸) and its nanodisc model (Skar-Gislinge *et al.*, 2010 ▸) to characterize the geometric parameters of the nanodisc. Similar to earlier efforts (Denisov *et al.*, 2004 ▸), *WillItFit* assumes a structural model that consists of components with simple geometric shapes. We examine the form factor of this kind of simplified structural model and correlate the structural parameters to the features observed in the scattering data. A similar analysis was also performed for the distance distribution function, which can be calculated directly from the electron-density distribution in the model. We then discuss the uniqueness of the data interpretation and the consequent implications for structural modelling of protein-loaded nanodiscs.

## 2. Materials and methods   

### 2.1. Nanodisc preparation   

#### 2.1.1. Membrane scaffolding proteins and lipids   

The membrane scaffolding proteins MSP1D1 and MSP1E3D1 were purchased from Sigma–Aldrich (St Louis, Missouri, USA). The N-terminal His tag was removed with TEV protease (Sigma–Aldrich) according to standard protocols. Briefly, MSPs at a concentration of ∼5 mg ml^−1^ in 25 m*M* Tris–HCl pH 8, 150 m*M* NaCl and 14 m*M* β-mercaptoethanol were treated with 1000 units of TEV protease per 3 mg of MSP. The reaction mixture was incubated at 303 K for 15 h. To check the extent of cleavage of His-tagged MSPs by TEV protease, an aliquot of the reaction mixture was removed and run on 4–20% gradient SDS-PAGE (Bio-Rad). A single faster-migrating band on SDS-PAGE confirmed successful cleavage of the N-terminal His tag. His-tagged cleaved MSPs, which eluted in the flow through, were quantified spectrophotometrically at 280 nm using molar extinction coefficients of 18 450 and 26 930 *M* ^−1^ cm^−1^ for MSP1D1 and MSP1E3D1, respectively. The 22 amino acid peptide and TEV protease were removed by nickel-affinity chromatography on a USB PrepEase Histidine-tagged Protein Purification cartridge according to the PrepEase standard procedure.

The lipids DMPC and 1,2-dipalmitoyl-*sn*-glycero-3-phosphocholine (DPPC) were purchased as powders from Sigma–Aldrich at >99% purity. The lipids were completely dissolved at a concentration of 50 m*M* in 20 m*M* Tris–HCl pH 7.4, 100 m*M* NaCl and 100 m*M* sodium cholate just prior to assembling the nanodisc.

#### 2.1.2. Self-assembly of nanodiscs   

Solutions of MSPs and detergent-solubilized lipids were mixed at the optimal molar ratio as determined by Sligar and co-workers (Denisov *et al.*, 2004 ▸; Bayburt & Sligar, 2010 ▸) (1:80 for MSP1D1:DMPC, 1:150 for MSP1E3D1:DMPC, 1:90 for MSP1D1:DPPC and 1:170 for MSP1E3D1:DPPC), making sure that the final sodium cholate concentration was between 12 and 40 m*M*. The reaction mixture was incubated for 60 min at the appropriate incubation temperature (310 K for DPPC and 298 K for DMPC). The reaction mixture was then placed inside a Spectra/Por 6-8K molecular weight cutoff dialysis membrane and dialysed against 1 l of 20 m*M* Tris–HCl pH 7.4, 100 m*M* NaCl at the incubation temperature. The dialysis buffer was replaced with fresh buffer four times over the course of a 48 h period. The fully assembled nanodiscs were removed from the dialysis membrane, centrifuged at 14 000 r min^−1^ for 15 min and concentrated in an Amicon Ultracel-30K centrifugal filter to 0.5 ml. The concentrated nanodisc solution was further centrifuged for 10 min at 14 000 r min^−1^ and then injected onto a 16/60 Superdex filtration column (GE Healthcare Life Sciences), previously equilibrated with 20 m*M* Tris–HCl pH 7.4, 150 m*M* NaCl and 0.5 m*M* EDTA. Column fractions containing nanodiscs were either pooled or kept separate, and concentrated in an Amicon Ultracel-30K centrifugal filter. The concentration of purified nanodiscs was determined from UV–vis absorption spectra using the MSP molar extinction coefficients.

### 2.2. Solution scattering data collection and model fitting   

X-ray scattering data were collected on National Synchrotron Light Source beamline X9 (Yang, 2013 ▸) at an X-ray energy of 13.5 keV. Samples containing fully assembled nanodiscs at concentrations ranging from 1 to 4 mg ml^−1^ were centrifuged for 30 min at 14 000 r min^−1^. They were then transferred to PCR tubes and measured using an in-house-built liquid-handling robot and a thermostated 0.9 mm quartz capillary flow cell. To minimize radiation damage, each sample was flowed through the flow cell at a rate of 0.7 µl s^−1^ during X-ray exposures. Three separate 30 s exposures were made for each sample. Data from the two-dimensional detectors were processed using a Python-based package developed at X9. The scattering data were radially integrated, averaged together and subtracted for buffer scattering following standard procedures. In all cases, the filtrate from the concentrator was used as the buffer blank. The one-dimensional data contain the scattered intensity as a function of the scattering vector magnitude *q* = (4π/λ)sinθ, where 2θ is the scattering angle and λ is the X-ray wavelength. The calculation of the distance distribution function, *P*(*r*), from the scattering data was done using the *ATSAS* software package (Franke *et al.*, 2017 ▸).

Geometric model fitting was performed using the program *WillItFit* (Pedersen *et al.*, 2013 ▸) and its nanodisc model. The number of adjustable parameters in the fit was kept to a minimum by incorporating molecular constraints in the model and by including best-fit values as previously determined by Skar-Gislinge & Arleth (2011 ▸) The height of the MSP belt and the number of hydration water molecules for the lipid head groups and MSP belts were all kept constant at 24 Å and 0, respectively. X-ray scattering lengths for the various components of the nanodisc (*e.g.* MSP belt, lipid head group, methylene chain and terminal methyl tail) were calculated from the chemical composition and the total number of electrons. The molecular volume of the MSP proteins was estimated from the protein molecular weight and an average protein partial specific volume of 0.73 cm^3^ g^−1^. The molecular volumes of the various lipid components were obtained from published values.

### 2.3. Scattering intensity calculation   

Following the *WillItFit* approach, we describe the nanodisc as an assembly of simple geometric shapes, consisting of the lipid bilayer, the protein belt and the membrane protein for a loaded disc. We also assume that the electron density of each component can be factorized into an in-plane part (parallel to the plane of the bilayer, *xy*) and an out-of-plane part (normal to the bilayer, along the *z* axis), as follows:
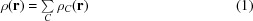
and 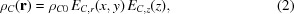
where *C* represents the lipid bilayer (Lp), the protein belt (BT) or the embedded membrane protein (MP). Each electron-density distribution is now expressed as an averaged electron density (ρ_*C*0_) and two dimensionless envelope functions defining the shape and density variation in the disc plane (*E*_*C*,*r*_) and along the disc normal (*E*_*C*,*z*_). Therefore, the scattering amplitude for each component of the nanodisc can be factorized into in-plane and out-of-plane parts as well: 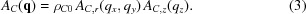
Each part can be calculated either numerically, using fast Fourier transform from maps of electron-density distribution in the *xy* plane and along the *z* axis, or analytically for simple geometric shapes.

The measured scattering intensity corresponds to the summed scattering amplitudes from each component, squared and isotropically averaged: 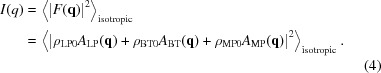
In polar coordinates, the isotropic average is given by
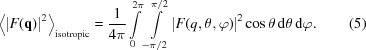



In order to trace the origin of the features in the scattering intensity, in the discussion below we present two-dimensional plots of the in-plane averaged intensity, defined as the square of the calculated amplitudes after the average over all possible in-plane (within the plane of the bilayer) orientations:
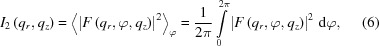
with *q_r_* = 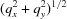 and tanφ = *q_y_*/*q_x_*. Numerically, this can be calculated for each *q*_*z*_ value from the factorized scattering amplitudes discussed above.

The cylindrical coordinates (*q_r_*, φ, *q_z_*) are related to the polar coordinates by *q_r_* = *q*cosθ and *q_z_* = *q*sinθ. Therefore the scattering intensity *I*(*q*) can be calculated from *I*_2_(*q_r_*, *q_z_*) using the following: 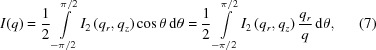
which is the two-dimensional isotropic average over θ on the *q_r_q_z_* plane of the quantity 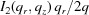.

## 3. Results and discussion   

### 3.1. Variability of the nanodisc structure   

It is well documented that the composition of the lipid–MSP mixture used for self-assembly is critical for obtaining nanodiscs of uniform size, with non-ideal stoichiometry leading to multiple structural species (Denisov *et al.*, 2004 ▸). However, even at the optimal mixing ratio, there is a finite size distribution for the nanodiscs (Denisov *et al.*, 2004 ▸; Marty *et al.*, 2014 ▸). Here, we characterized the size distribution of the MSP1E3D1:DMPC nanodiscs at the nominally optimal composition using X-ray scattering.

**Figure 1:**
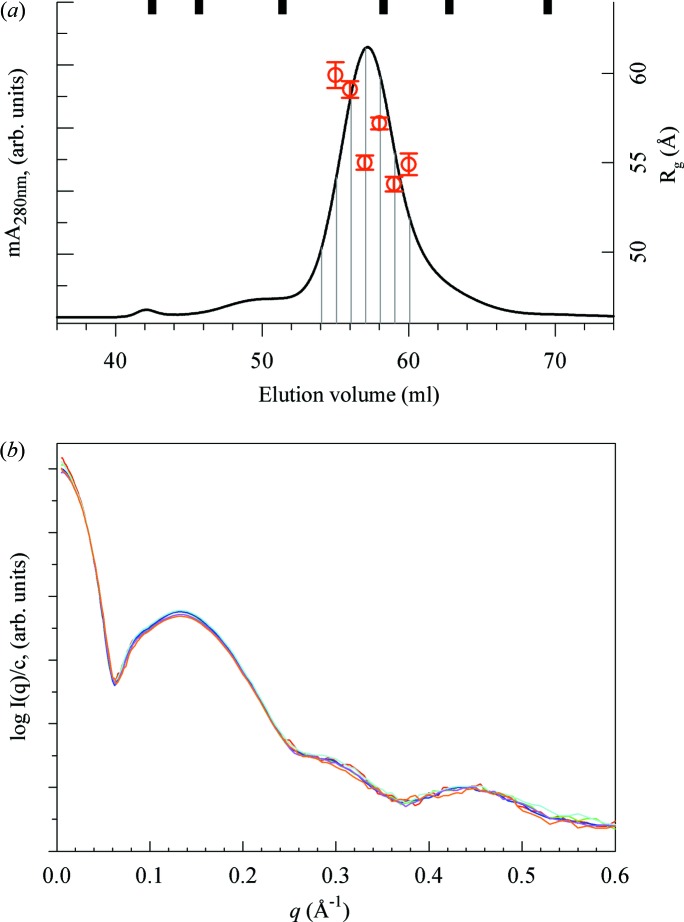
(*a*) The size-exclusion chromatography profile of MSP1E3D1:DMPC nanodiscs assembled under optimal stoichiometry and the scattering data from different fractions, as indicated by the vertical drop lines. Also plotted are the radii of gyration for each fraction (red symbols). The markers on top of the chromatogram denote the elution times of molecular weight standards (from left to right: the void volume, and 669, 443, 200, 150 and 66 kDa molecular masses). The flow rate was 1 ml min^−1^. (*b*) The scattering data, normalized for concentration. Data from fractions 55–60 min are coloured red, green, blue, cyan, magenta and orange, respectively.

We used size-exclusion chromatography (SEC) to separate the potentially different structural species and collected scattering data on each SEC fraction. The resulting chromatogram (Fig. 1 ▸ *a*) shows one predominant peak corresponding to correctly assembled nanodiscs, although a small, broader, peak that elutes earlier is clearly visible. This peak, which is greatly enhanced when the stoichiometry is incorrect, corresponds to larger lipid–MSP particles. It is important to point out that, when working with membrane-protein-loaded nanodiscs, the loaded normal discs could potentially co-elute with this unloaded contaminant, thereby compromising the purity of the sample.

The concentration-normalized X-ray scattering data corresponding to different SEC fractions of the MSP1E3D1:DMPC nanodiscs are shown in Fig. 1 ▸(*b*). The radius of gyration obtained based on a Guinier approximation is consistent with the retention time, *i.e.* larger particles have shorter retention times. This suggests that the peak width in the chromatogram is not limited by the intrinsic resolution of SEC. Therefore there is a small but definitive size variation of the nanodiscs, even within the same peak in the chromatogram. The size variation is also supported by the *WillItFit* results on the individual scattering profile (Table S1 in the supporting information). This result is consistent with a similar study analysing the size of 1-palmitoyl-2-oleoyl-*sn*-glycero-3-phosphocholine (POPC) nanodiscs (Skar-Gislinge, 2014 ▸), where the forward scattering intensity *I*_0_ after concentration normalization was used as the metric.

Given that the nanodiscs are self-assembled from soft components, it is not surprising that there are variations among the assembled structures. This is supported by evidence from other experimental methods. For instance, Denisov *et al.* (2004 ▸) reported a ±3% size distribution on the basis of scintillation counting, while Marty *et al.* (2014 ▸) reported a standard deviation of lipid count per disc of up to ±4% on the basis of mass spectroscopy. On the other hand, the two protein belts around the lipid bilayer are stabilized by highly specific interactions between them (Bibow *et al.*, 2017 ▸) and expected to hold a constant circumference. Therefore the variation in the number of lipids will necessarily give rise to some variation in the shape of the nanodisc, which will probably increase the spread of the measured radius of gyration from that expected for the size variation alone.

Despite the variation in the Guinier region, the scattering intensity at *q* > 0.3 Å^−1^ appears fairly constant, even though the signal-to-noise ratio is decreasing. This is consistent with the assessment (to be discussed below in §3.2) that the features in the scattering intensity within this *q* range are mainly due to the bilayer structure, which is expected to remain the same across the SEC fractions.

**Figure 2:**
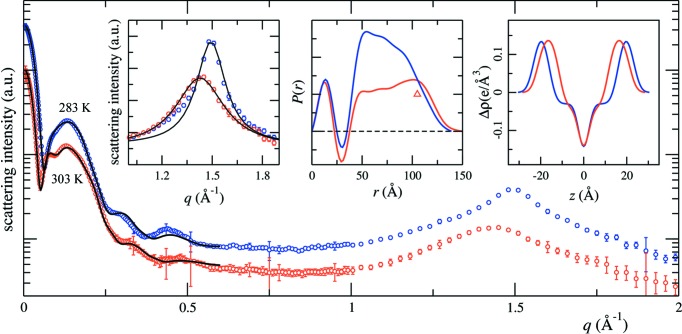
Structural changes in the MSP1E3D1:DMPC nanodisc across the phase transition temperature of the DMPC bilayer. The scattering data were collected at 283 K (blue symbols) and 303 K (red symbols). The solid black lines show the *WillItFit* results (up to *q* = 0.6 Å^−1^). The left inset replots the wide-angle portion of the scattering data (symbols) on a linear intensity scale, compared with two Lorentzian functions (solid lines) as references for peak symmetry. The 283 K data (blue) show a higher intensity on the low-*q* side of the peak. The pair distance distribution functions (middle inset, same colour code) derived from the data show a larger maximum dimension and the protein belt peak (indicated by the triangle, as discussed in §3.3) shifting to a larger distance at 303 K. The right inset shows the electron-density profile of the bilayers based on the *WillItFit* results (Table S2 in the supporting information).

The soft nanodisc structure can accommodate changes after the nanodisc is fully assembled, for instance when the lipid bilayer is driven through its main phase transition. This transition has been reported to be reversible and not alter the composition of the nanodisc (Shaw *et al.*, 2004 ▸; Skar-Gislinge *et al.*, 2010 ▸), yet there are significant structural changes across this transition, as shown by the scattering data collected from the MSP1E3D1:DMPC nanodiscs at 283 and 303 K (Fig. 2 ▸). Model fitting results using *WillItFit* show that the bilayer structure has changed (Fig. 2 ▸, inset) across the transition, as expected. In addition, the disc size and shape have changed (Table S2 in the supporting information).

### 3.2. Common features in the small-angle scattering data   

Following the approach described in §2.3, we calculated the nanodisc scattering intensity using a geometric model similar to the one used by *WillItFit*, but instead of directly presenting the final results of the isotropically averaged scattering intensity *I*(*q*), we examined the intermediate steps in this calculation and explored how the signatures due to various structural features, such as the bilayer density profile, might be preserved in the final result.

**Figure 3:**
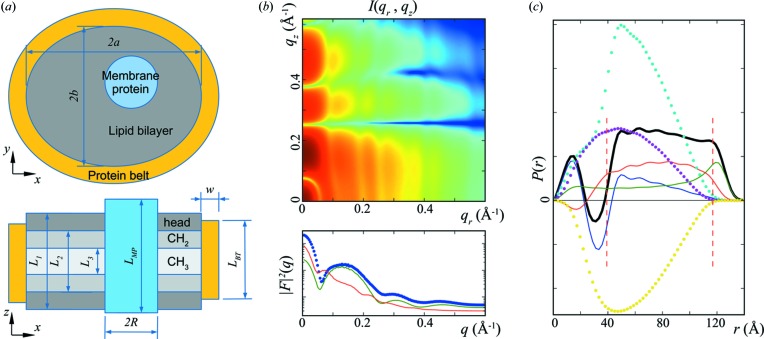
(*a*) The geometric model used and definitions of the relevant parameters. (*b*), (*c*) A breakdown of the scattering intensity and the *P*(*r*) function for a lipid nanodisc, based on the model shown in panel (*a*). The scattering intensity of panel (*b*) is first represented as a two-dimensional function *I*(*q*_*r*_, *q*_*z*_) (upper panel) and subsequently converted into an isotropic average (blue symbols in the lower panel), as described in the text. The geometric parameters are based on the *WillItFit* results for the 55 min fraction data presented in Fig. 1 ▸. For comparison, the isotropic average of the contributions from the lipid bilayer alone (green line) and the protein belt alone (red line) are also shown. Note that a small constant has been added to the calculated intensity to match the experimental data. The two-dimensional scattering amplitude maps for the individual components and the corresponding one-dimensional scattering profiles are presented in the supporting information (Figs. S1 and S2). The breakdown of the distance distribution function *P*(*r*) (thick black line) is shown in panel (*c*), as calculated from the same geometric model. The contributions from the individual components are also shown: lipid heads (cyan dots), lipid chains (magenta dots), the cross-correlation between the lipid heads and lipid tails (yellow dots), the lipid bilayer as a whole (blue line), the protein belt (green line), and the cross correlation between the lipid bilayer and the protein belt (red line). Note that these curves have been smoothed using a low-pass filter to eliminate artefacts due to the discrete grid used to represent the continuous electron-density distribution of the nanodisc. For reference, the vertical red dashed lines indicate the head-to-head distance of the bilayer [(*L*_1_ + *L*_2_)/2] and a characteristic length {[(2*a*)^2^ + (*L*_BT_)^2^]^1/2^} to represent the protein belt.

The geometric parameters that describe the nanodisc structure are defined in Fig. 3 ▸(*a*). All shapes are based on ellipses in the *xy* plane and boxes (rectangles) along the *z* axis. It is therefore computationally economical to make use of the factorized analytical form of the Fourier transform of these density distributions. For an ellipse with semi-major and semi-minor axes *a* and *b*, the in-plane part of the scattering amplitude is 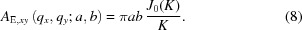
Here, *J*_0_ is a Bessel function and *K* = [(*q_x_a*)^2^ + (*q_y_b*)^2^]^1/2^ is simply the result of coordinate substitution in the Fourier transform.

In order to simulate realistic gradual electron-density changes, transitions between constant densities are represented by an error function of width σ (a Gaussian function convoluted with a step function). This finite value of σ, which can be likened to a surface roughness and is also employed in the *WillItFit* nanodisc model (Skar-Gislinge & Arleth, 2011 ▸), results in an exponential decay in the scattering amplitude: 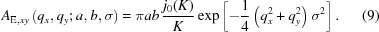
Along the *z* axis, the scattering amplitude of a box electron-density distribution, centred at *z* = 0, with a width *L* and roughness σ, is
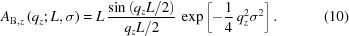
The partial scattering amplitude of the nanodisc component can then be constructed from these basic amplitudes. Specifically, for the protein belt, 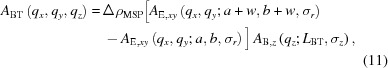
where Δρ_MSP_ is the electron-density contrast of the protein belt against water.

For the lipid bilayer, its density profile along the *z* axis can be described as three sublayers of different electron-density contrast against water (ρ_head_ for the head groups, ρ_core_ for the methylene chains and ρ_methyl_ for the terminal methyl groups) and each sublayer can take on distinct roughness values, σ_*z*1_, σ_*z*2_ and σ_*z*3_, as we will discuss below. The scattering amplitude of the bilayer is therefore 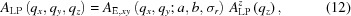
where the the *q*_*z*_ dependence of the scattering amplitude is given by 
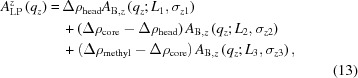



For simplicity, in this study we represent the membrane protein as a simple cylinder of radius *R* and height *L*_MP_. The membrane protein occupies a volume that is otherwise part of the bilayer structure. Its contribution to the scattering amplitude should therefore account for the amplitude due to this displaced bilayer structure, 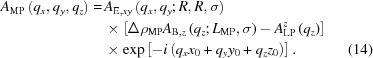
The phase shift at the end of this equation is present when the centre of the membrane protein is located at an offset vector 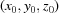 from the geometric centre of the empty nanodisc. Also for simplicity, we do not account for the hydration water. This contribution to the scattering intensity is expected to be small owing to the relatively large size of the nanodisc.

The synthesis of the final scattering intensity from the contributions of the individual components is shown in Fig. 3 ▸(*b*), and in Figs. S1 and S2 in the supporting information. The combined two-dimensional form factor of the nanodisc, 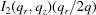, shows that the interference of the scattering amplitudes due to individual components results in a multitude of local peaks and valleys. However, most features that appear in the final isotropically averaged scattering intensity can be traced back to the intensity profiles along the *q*_*r*_ and *q*_*z*_ axes, 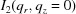 and 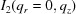 (Fig. S2*a* in the supporting information), which correspond to the averaged electron-density distribution projected onto the *xy* plane and onto the *z* axis, respectively. This is simply because the scattering intensity drops off with increasing *q*_*r*_ or *q*_*z*_ values. Therefore the intensity at *q*_*r*_ = 0 or *q*_*z*_ = 0 would make the most significant contribution to the isotropic average [equation (7)]. The position of the first valley at *q* ≃ 0.1 Å^−1^ is influenced by both the in-plane structure of the disc and the bilayer structure. At higher *q* values (*q* > 0.2 Å^−1^), the profile of the scattering intensity appears to be dominated by features due to the bilayer structure. This may simply be because the magnitude drop-off in *q*_*z*_ is slower for the bilayer structure (see Fig. S2*b* in the supporting information) and there are no prominent features in 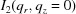 in this *q* range.

**Figure 4:**
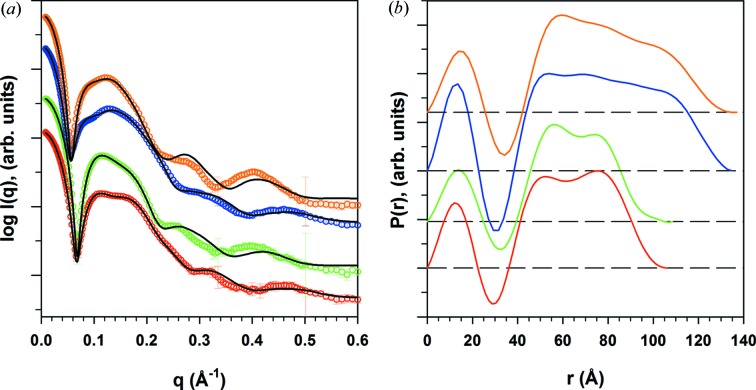
(*a*) Scattering data for four different types of nanodisc construct. Nanodiscs were assembled using two types of lipid and two types of MSP construct: from bottom to top, MSP1D1:DMPC (red open circles), MSP1D1:DPPC (green open circles), MSP1E3D1:DMPC (blue open circles) and MSP1E3D1:DPPC (orange open circles). The scattering curves are plotted on an arbitrary scale and are displaced vertically for clarity. The solid black lines show the results from *WillItFit*. The fitting parameters are listed in Table S3 in the supporting information. (*b*) Pair distance distribution functions derived from the SAXS data in panel (*a*), shown with the same colour key. The *P*(*r*) curves are displaced vertically for clarity. The disc size difference between MSP1D1 and MSP1E3D1 is apparent.

The association of these features with the nanodisc structure is consistent with the *WillItFit* results of the MSP1E3D1:DMPC nanodisc phase transition data (Fig. 2 ▸): the data at *q* > 0.3 Å^−1^ indicate a bilayer structure change, while low-*q* data indicate a disc size or shape change. Further support of this interpretation can be found in the scattering data shown in Fig. 4 ▸(*a*), collected from four different types of nanodisc construct of two types of lipid (DMPC and DPPC) and two MSPs of different length (MSP1D1 and MSP1E3D1). For nanodiscs constructed from the same MSP, the first valley in the scattering data appears at approximately the same location. On the other hand, the subsidiary peaks at *q* > 0.3 Å^−1^ appear to depend on the type of lipid. Of course, the size and shape of the nanodisc can also affect the scattering intensity of the nanodisc in this *q* range through the *q*_*r*_ dependence of the bilayer scattering amplitude in equation (12). However, this effect is greatly diminished when the disc shape is not circular or has a finite size distribution, as we will discuss below.

### 3.3. Common features in the distance distribution function   

From the geometric model of the nanodisc, we can also calculate the distance distribution function, *P*(*r*). In order for this calculation to be computationally manageable, we represent the continuous electron-density distribution of the nanodisc with a lattice, with typically 50–70 nodes in each direction. The charge at each node of the lattice corresponds to the integrated electron density within the voxel occupied by this node. Once the charge-weighted distance histogram is calculated, we apply a low-pass filter to eliminate artefacts due to the finite spacing of the lattice to give the final *P*(*r*) function.

In order to trace the origin of the features in *P*(*r*), we calculate the contribution from components of the nanodisc as well (Fig. 3 ▸ *c*), due, respectively, to the lipid bilayer alone, the protein belt alone, and the cross correlation between the lipid bilayer and the protein belt. The lipid bilayer can be further analysed by parts (dotted lines). One might expect the negative valley in *P*(*r*) to be due to the correlation between the lipid head groups and chains, which exhibit electron densities of opposite signs. In reality, however, this valley is really the result of the superposition of several contributions, and its position cannot be simplistically attributed to any single structure parameter. This may explain why the *P*(*r*) functions corresponding to the same lipid species in Fig. 4 ▸(*b*) do not exhibit a valley at the same location. On the other hand, the peak in the *P*(*r*) function near the maximum dimension clearly comes from the self-correlation of the protein belt, which is a ring of high electron density.

With this in mind, we note that, in the MSP1E3D1:DMPC nanodisc phase transition data (Fig. 2 ▸), the distance distribution function at 303 K exhibits a peak near the maximum dimension (indicated by the red triangle). Similar behaviour of the *P*(*r*) function during the bilayer phase transition was also observed in nanodiscs that contain 1,2-dilauroyl-sn-glycero-3-phosphocholine and POPC (Skar-Gislinge *et al.*, 2010 ▸). The *P*(*r*) function is in effect a histogram of intramolecular distances within the molecular envelope. For an enclosed protein belt of a given length, the highest probability of the same intra­molecular distance occurs when the belt forms a circle. Accordingly, the peak in the *P*(*r*) function is expected to be most prominent at a distance that equals the diameter of the circle. On the other hand, this peak would be diminished if the chain were to take on an elliptical or any other non-circular shape. Therefore these data suggest that the nanodisc shape is likely to be more circular in the fluid phase than in the gel phase. This certainly makes physical sense. In the fluid phase, the bilayer is expected to be more flexible and the shape of the nanodisc is probably determined by the elastic properties of the protein belt. In the gel phase the lipid bilayer becomes thicker, and consequently the bilayer area is smaller, as confirmed by the smaller maximum dimension in *P*(*r*). Under the constraint of constant circumference, as discussed in §3.1, the nanodisc would adopt more a distorted shape to accommodate the smaller area.

### 3.4. Wide-angle scattering data   

The organization of lipid chains in the bilayer results in a peak in the scattering data near ∼1.5 Å^−1^, which is clearly seen in the data presented in Fig. 2 ▸. At 303 K (red symbols in the inset) this peak is symmetric, consistent with a fluid-phase bilayer structure. In contrast, below the phase transition temperature this peak shifts to higher *q* and becomes sharper and asymmetric, with a shoulder on the low-*q* side (red symbols in the inset). This suggests that the lipid chains are tilted from the bilayer normal direction, as is well known in the gel phase (Smith *et al.*, 1988 ▸; Yang & Fukuto, 2005 ▸). The chain tilt breaks the symmetry and may further favour a non-circular disc shape.

The width of the gel-phase chain packing measured from multiple bilayers is often limited by the instrument resolution (see *e.g.* Yang & Fukuto, 2005 ▸). This peak width in the gel-phase nanodisc data is ∼0.2 Å^−1^, well above the instrument resolution. A rough estimate (2π/Δ*q*) of the domain size of the hexagonal lattice formed by the lipid chains gives ∼30 Å, which is of the same order of magnitude as, but smaller than, the size of the lipid bilayer enclosed in the nanodisc. This may be an indication that, at low temperatures, not all of the lipid bilayer has transitioned to the gel phase, at least not to the same gel phase as observed in bulk bilayers. This type of structural inhomogeneity within the nanodisc bilayer has been discussed in computer simulation studies (Siuda & Tieleman, 2015 ▸). Denisov *et al.* (2005 ▸) also speculated the same, on the basis of the larger-than-expected area per lipid derived from fitting the X-ray scattering data. However, the area per lipid derived from our data appears to be normal (Table S2 in the supporting information).

### 3.5. Modelling of the nanodisc structure   

Structural modelling for solution scattering in general faces the issues of accuracy and uniqueness, for instance in the case of *ab initio* shape modelling as discussed by Volkov & Svergun (2003 ▸). The modelling algorithm may not converge easily if there are a large number of free parameters. On the other hand, if there are too few parameters the resulting model may miss important structural features or give misleading results. We argue that the inclusion of scattering data at *q* > 0.3 Å^−1^ can effectively improve confidence in the nanodisc model, as we discuss below within the context of the *WillItFit* nanodisc model.

#### 3.5.1. Lipid bilayer density profile   

In the analysis of scattering data, lipid bilayers are often described as a stack of homogeneous layers, each with a different electron density. As discussed in §3.2, a roughness value is introduced as a means of describing a continuous transition of the electron density near interfaces. The *WillItFit* nanodisc model assumes a single roughness value for all interfaces in the model (Pedersen *et al.*, 2013 ▸). This permits the scattering intensity to be calculated from a model with sharp interfaces by simply multiplying the intensity by a single Gaussian function. This simplification limits the range of variations in the lipid bilayer structure that may be used to fit the data. Specifically, it is well known that the electron-density profile for lipid membranes in the gel phase exhibits a plateau in the methylene chain region. In this case, describing the bilayer structure using a single roughness value is typically adequate. However, this plateau is generally absent when the membrane is in the liquid crystalline phase. It then becomes impractical to describe the bilayer electron-density profile using a single value of interfacial roughness.

**Figure 5:**
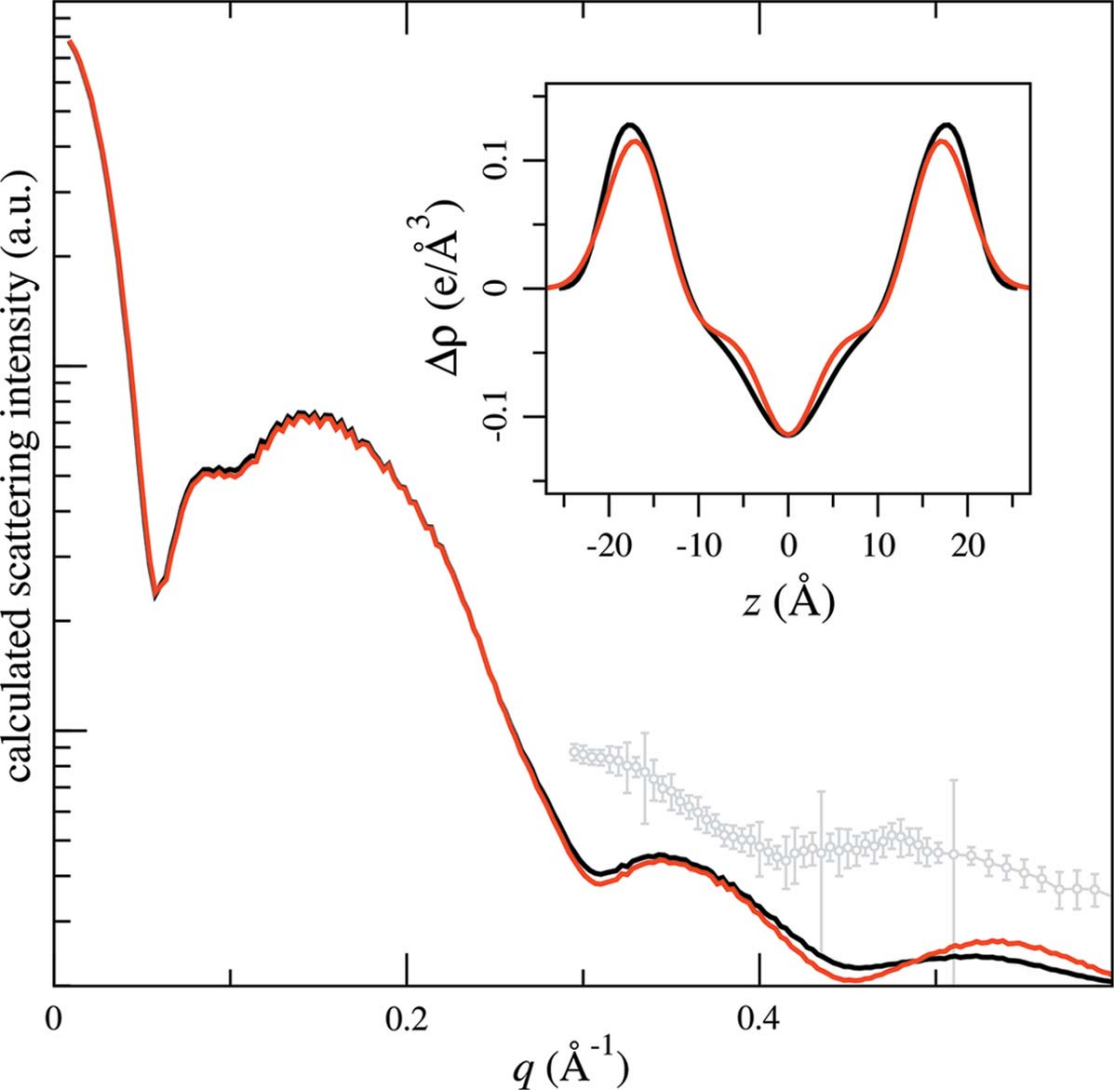
Different lipid bilayer structures (inset) could produce very similar scattering intensities, especially at *q* < 0.3 Å^−1^ (main image). The scattering intensity shown in black is based on a published DMPC lipid bilayer structure in the fluid phase (Toppozini *et al.*, 2012 ▸). We fitted these data using *WillItFit* to arrive at a different bilayer density profile, and the corresponding scattering intensity is shown in red. All parameters used for the calculations are listed in Table S4 in the supporting information. Also shown in grey symbols as a reference is a subset of the MSP1E3D1:DMPC nanodisc 303 K data from Fig. 2 ▸.

To test the influence of the lipid density profile on the scattering intensity, we calculated the scattering intensity on the basis of a published structure of a fluid DMPC bilayer at 303 K (Toppozini *et al.*, 2012 ▸). We then fitted the simulated SAXS data using *WillItFit* (Fig. 5 ▸). As expected, the resulting lipid density profile based on a single roughness value looks quite different from the input, which is represented by three different roughness values (Fig. 5 ▸, inset, and Table S4 in the supporting information).

As discussed in §3.2, the scattering data within the *q* range of 0.3–0.6 Å^−1^ can be attributed to the lipid bilayer structure. For reference, we also replotted part of the 303 K MSP1E3D1:DMPC nanodisc data in Fig. 5 ▸. The experimental data are substantially different from our synthesized data, suggesting that the assumed fluid-phase lipid bilayer structure still does not reflect the actual structure. It may be an effective strategy to obtain accurate parameters to describe the bilayer structures if higher weights are preferentially assigned to the high-*q* data in the fitting process.

#### 3.5.2. Shape of the nanodisc   

The *WillItFit* nanodisc model assumes that the nanodiscs are elliptical instead of circular. It has been argued (Skar-Gislinge *et al.*, 2010 ▸) that the elongated disc shape is a consequence of entropy favouring nanodiscs that are not fully loaded with lipid molecules. The lack of high-frequency oscillations in our scattering data in the range 0.3–0.6 Å^−1^ lends support to this assumption.

From the practical standpoint of curve fitting, a consequence of an elliptical disc shape is the reduced depths of the valleys observed in the *q*_*r*_ dependence of the form factor. The form factor of a circular disc exhibits sharp valleys in *q*_*r*_. These valleys become filled in in the in-plane isotropic average [equation (6)] for an elliptical disc since the positions of these valleys are dependent on the in-plane direction, leaving the intensity at high *q* dominated by oscillations that are due to the bilayer structure (intensity variation near the *q*_*z*_ axis on the two-dimensional intensity map). In contrast, a circular disc structure of radius *R* would produce fringes at a periodicity of ∼π/*R* (approximate spacing between the zeroes of the Bessel function *J*_0_) at high *q*. These fringes are not observed in our data. It is interesting to point out that an irregular disc shape results in complex scattering amplitudes with axial dependence, and consequently the valleys in the disc form factor are not filled in as effectively as with an elliptical disc (Fig. S3 in the supporting information).

In principle, a finite size distribution of circular discs can also wash out the high-*q* fringes owing to the disc size. However, this would require a fairly wide size distribution. For instance, with an average radius of 40 Å, a size difference of ∼5 Å is needed to produce a phase reversal of the fringes (peak *versus* valley in the intensity) at *q* ≃ 0.3 Å^−1^. That is a size distribution of more than 20%, which is not realistic for nanodiscs that are prepared following optimized protocols (see §3.1).

### 3.6. Comments on structural modelling of nanodisc-embedded membrane protein structure   

As discussed in the *Introduction*, for the solution scattering community the purpose of structural characterization of empty nanodiscs is to use it as an input in the modelling of membrane proteins embedded in the nanodisc. Unlike buffer scattering in the measurements of soluble proteins, the scattering contribution of the empty nanodisc cannot be simply subtracted out [see equation (4)]. Furthermore, in order to make use of the empty nanodisc structure in modelling, the scattering contribution from the membrane protein should be calculated from the electron-density contrast of the membrane protein against the lipid bilayer according to equation (14). Therefore, ambiguity in the bilayer structure could lead to a different model for the membrane protein from a given set of scattering data. The position of the embedded membrane protein within the nanodisc can also influence the scattering intensity of the loaded nanodisc. Varying the location of the membrane protein effectively alters the phase shift between the scattering amplitudes of the membrane protein and the empty nanodisc [equation (14)] and consequently the total scattering intensity.

**Figure 6:**
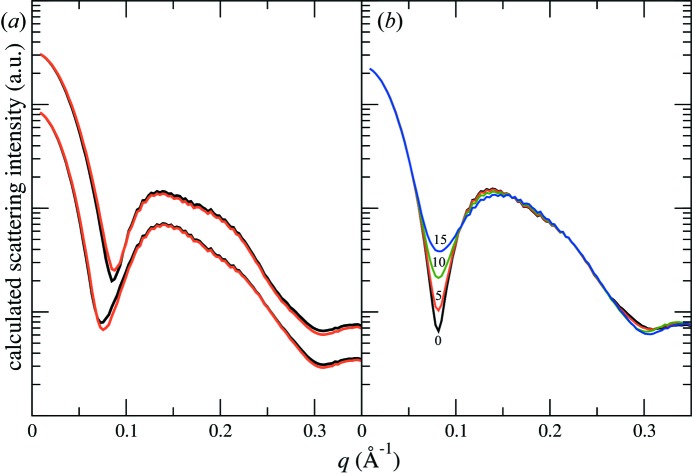
Calculated scattering intensity from membrane-protein-loaded nanodiscs. The membrane protein is represented by a cylinder 44.3 Å in height. (*a*) Ambiguity in the bilayer structure discussed in §3.5.1 can lead to different scattering intensities expected for the same membrane protein located at the centre of the nanodisc. The calculations were done for two membrane protein diameters, 25 Å (bottom) and 45 Å (top). The empty nanodisc structures are taken from Fig. 5 ▸ with the same colour code. The intensities for the two different protein sizes are offset for clarity. (*b*) Varying the position of the membrane protein (35 Å in diameter) within the nanodisc results in dramatic changes in the scattering intensity. The same fluid-phase nanodisc structure (black lines) in panel (*a*) is used in this calculation. The membrane protein is systematically shifted along the major axis of the elliptical nanodisc from the centre by 0 (black), 5 Å (red), 10 Å (green) and 15 Å (blue).

We demonstrate these effects using the two empty nanodisc structures discussed in §3.5.1. For simplicity, we represent the membrane protein as a right circular cylinder 44.3 Å in height and with a uniform excess electron density of Δρ_MP_ = 0.106 e Å^−3^. The scattering intensity is calculated for membrane proteins positioned at the centre of the nanodisc for different cylinder diameters (25 and 45 Å, Fig. 6 ▸ *a*). Differences in the scattering intensity are clearly more pronounced compared with the empty nanodiscs (Fig. 5 ▸), even at *q* < 0.3 Å^−1^, where the two empty discs show virtually identical scattering intensity. More dramatic changes are observed for the same membrane protein of 35 Å diameter at different offsets along the long axis of the elliptical disc (0, 5, 10 and 15 Å, Fig. 6 ▸ *b*). Ideally, the uncertainty in the membrane protein position should be removed during the experimental design stage, by selecting an MSP that sufficiently constrains the membrane protein motion. Otherwise a significantly more complex model may be needed to take into account all possible protein positions.

## 4. Conclusions   

Lipid nanodiscs are fairly complex structures. A large number of structural parameters are needed to describe them. The number of free parameters during model fitting can be reduced by applying appropriate constraints, as is done in the *WillItFit* nanodisc model. However, there may still be multiple plausible interpretations for the same scattering data. Precautions should be taken to eliminate ambiguities. During the experimental design stage, the length of the protein belt should be chosen so as to limit the motion of the membrane protein within the nanodisc. During sample preparation, rigorous quality control is critical to ensure uniform nanodisc size and shape. In-line size-exclusion chromatography has become widely available at synchrotron beamlines and should be utilized in measurements on nanodiscs. During data analysis, while it is important to apply constraints when modelling the nanodisc structure, it is also necessary to leave enough flexibility (*e.g.* different roughness values to describe the electron-density transitions) to describe the structure accurately. Obtaining additional inputs from the same structures, either by extending to higher values of scattering vectors as we have shown, or by combining X-ray and neutron scattering with contrast variation on the same structures (see *e.g.* Skar-Gislinge *et al.*, 2010 ▸, 2015 ▸), will further improve the accuracy of the structural model. It should also be recognized that nanodiscs are built from flexible components and structural variations may be inevitable.

## Supplementary Material

Figures showing calculations discussed in the text, and tables of fitting parameters. DOI: 10.1107/S1600576717018441/vg5078sup1.pdf

## References

[bb1] Bayburt, T. H., Grinkova, Y. V. & Sligar, S. G. (2002). *Nano Lett.* **2**, 853–856.

[bb2] Bayburt, T. H. & Sligar, S. G. (2010). *FEBS Lett.* **584**, 1721–1727. 10.1016/j.febslet.2009.10.024 PMC4758813 19836392

[bb3] Bibow, S., Polyhach, Y., Eichmann, C., Chi, C. N., Kowal, J., Albiez, S., McLeod, R. A., Stahlberg, H., Jeschke, G., Güntert, P. & Riek, R. (2017). *Nat. Struct. Mol. Biol.* **24**, 187–193. 10.1038/nsmb.3345 28024148

[bb4] Denisov, I. G., Grinkova, Y. V., Lazarides, A. A. & Sligar, S. G. (2004). *J. Am. Chem. Soc.* **126**, 3477–3487. 10.1021/ja0393574 15025475

[bb5] Denisov, I. G., McLean, M. A., Shaw, A. W., Grinkova, Y. V. & Sligar, S. G. (2005). *J. Phys. Chem. B*, **109**, 15580–15588. 10.1021/jp051385g PMC2518645 16852976

[bb6] Denisov, I. G. & Sligar, S. G. (2016). *Nat. Struct. Mol. Biol.* **23**, 3195. 10.1038/nsmb.3195 PMC8934039 27273631

[bb7] Franke, D., Petoukhov, M. V., Konarev, P. V., Panjkovich, A., Tuukkanen, A., Mertens, H. D. T., Kikhney, A. G., Hajizadeh, N. R., Franklin, J. M., Jeffries, C. M. & Svergun, D. I. (2017). *J. Appl. Cryst.* **50**, 1212–1225. 10.1107/S1600576717007786 PMC5541357 28808438

[bb8] Marty, M. T., Zhang, H., Cui, W., Gross, M. L. & Sligar, S. G. (2014). *J. Am. Soc. Mass Spectrom.* **25**, 269–277. 10.1007/s13361-013-0782-y PMC3918181 24353133

[bb9] Pedersen, M. C., Arleth, L. & Mortensen, K. (2013). *J. Appl. Cryst.* **46**, 1894–1898.

[bb10] Sanders, C. R. & Landis, G. C. (1995). *Biochemistry*, **34**, 4030–4040. 10.1021/bi00012a022 7696269

[bb11] Seddon, A. M., Curnow, P. & Booth, P. J. (2004). *Biochim. Biophys. Acta*, **1666**, 105–117. 10.1016/j.bbamem.2004.04.011 15519311

[bb12] Shaw, A. W., McLean, M. A. & Sligar, S. G. (2004). *FEBS Lett.* **556**, 260–264. 10.1016/s0014-5793(03)01400-5 14706860

[bb13] Siuda, I. & Tieleman, D. P. (2015). *J. Chem. Theory Comput.* **11**, 4923–4932. 10.1021/acs.jctc.5b00668 26574280

[bb17] Skar-Gislinge, N. (2014). PhD thesis, University of Copenhagen, Denmark.

[bb14] Skar-Gislinge, N. & Arleth, L. (2011). *Phys. Chem. Chem. Phys.* **13**, 3161–3170. 10.1039/c0cp01074j 21152549

[bb15] Skar-Gislinge, N., Kynde, S. A. R., Denisov, I. G., Ye, X., Lenov, I., Sligar, S. G. & Arleth, L. (2015). *Acta Cryst.* D**71**, 2412–2421. 10.1107/S1399004715018702 PMC4667284 26627649

[bb16] Skar-Gislinge, N., Simonsen, J. B., Mortensen, K., Feidenhans’l, R., Sligar, S. G., Lindberg Møller, B., Bjørnholm, T. & Arleth, L. (2010). *J. Am. Chem. Soc.* **132**, 13713–13722. 10.1021/ja1030613 PMC4120756 20828154

[bb18] Smith, G. S., Sirota, E. B., Safinya, C. R. & Clark, N. A. (1988). *Phys. Rev. Lett.* **60**, 813–816. 10.1103/PhysRevLett.60.813 10038659

[bb19] Toppozini, L., Armstrong, C. L., Barrett, M. A., Zheng, S., Luo, L., Nanda, H., Sakai, V. G. & Rheinstädter, M. C. (2012). *Soft Matter*, **8**, 11839–11849.

[bb20] Tribet, C., Audebert, R. & Popot, J.-L. (1996). *Proc. Natl Acad. Sci. USA*, **93**, 15047–15050. 10.1073/pnas.93.26.15047 PMC26353 8986761

[bb21] Volkov, V. V. & Svergun, D. I. (2003). *J. Appl. Cryst.* **36**, 860–864.

[bb22] Yang, L. (2013). *J. Synchrotron Rad.* **20**, 211–218. 10.1107/S0909049512048984 23412476

[bb23] Yang, L. & Fukuto, M. (2005). *Phys. Rev. E*, **72**, 010901. 10.1103/PhysRevE.72.010901 16089928

