# Supplementary material for: Interpretation of solution scattering data from lipid nanodiscs
Source: J Appl Crystallogr. 2018 Feb 1;51(Pt 1):157–66. doi: 10.1107/S1600576717018441 (PMC5822991; doi:10.1107/S1600576717018441)

JOURNAL OF
APPLIED
CRYSTALLOGRAPHY

Volume 51 (2018)

Supporting information for article:

Interpretation of solution scattering data from lipid nanodiscs

Vito Graziano, Lisa Miller and Lin Yang

Supplementary Information

1. Further break-down of nanodisc form factor

Figure S1 Partial form factors (squared) of the patch of lipid bilayer (a), the protein belt (b), and the membrane protein (c). Each form factor is the product of an in-plane (q_r-dependent) part and an out-of-plane (q_z-dependent) one.

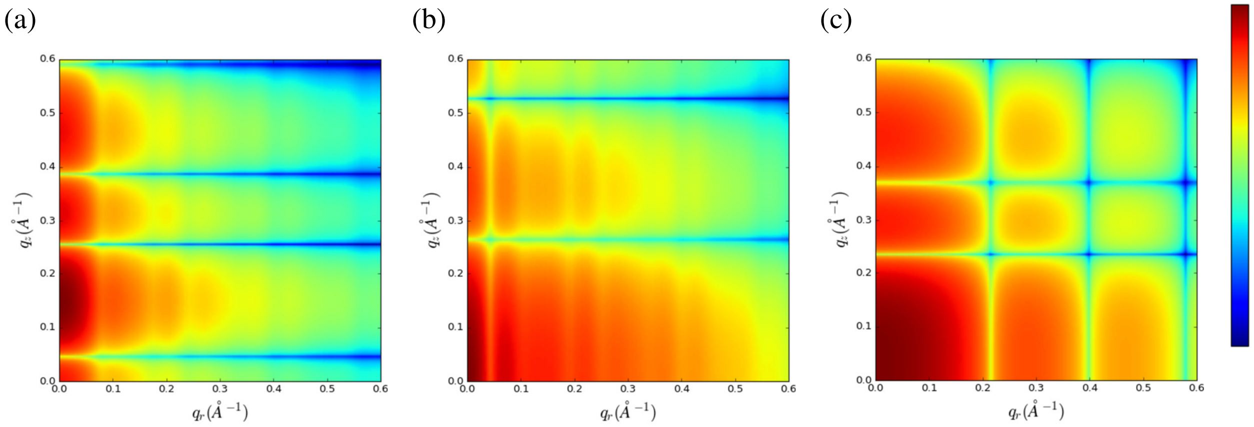

Figure S2 The total scattering intensity (a), the partial form factor of the lipid bilayer (b), and the partial form factor of the protein belt (c). In each case, the isotropically averaged value is compared to the profile along the q_r - and q_z - axes on the 2D intensity map shown above and in Figure 4. Interestingly, the high q_z features from the bilayer structure appears to survive the isotropic average better, since its value drops off more slowly as q_z increases.

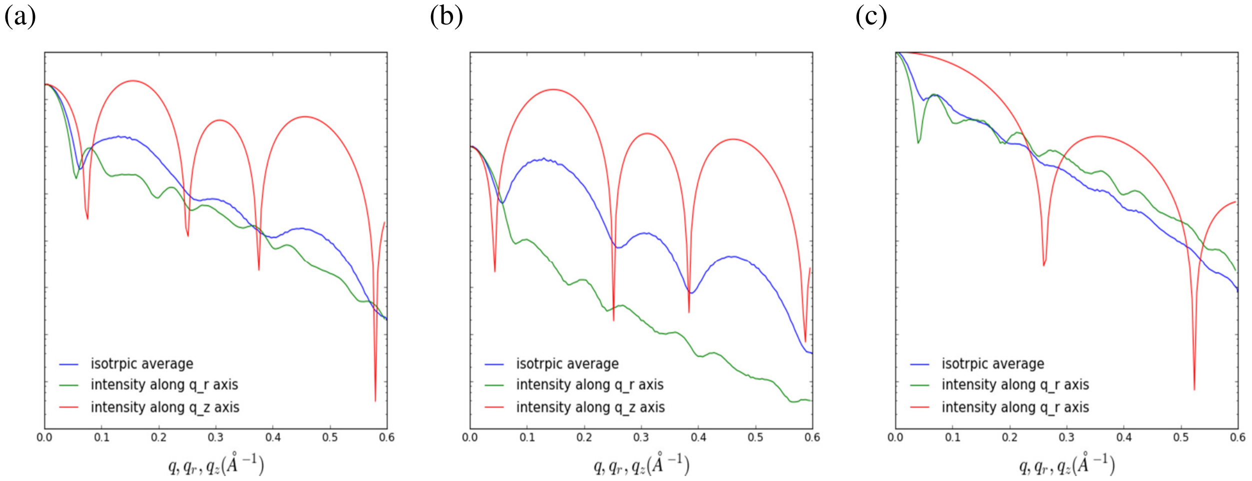

2. WillItFit results

Not all parameters are listed (e.g. the constant background and the scaling factors). The parameters with a ± are directly determined from the fit (the uncertainty is given by WillItFit); the rest are derived by molecular constraints on the model. N_{lipids}, number of lipids in the nanodisc; A, area per lipid head group; R_1, semi-major axis; R_2, semi-minor axis; $H_{bilayer}$, height of lipid bilayer; H_{core}, height of lipid core; H_{methyl}, height of methyl layer; W_{belt}, MSP thickness; $\Delta\rho_{head}$, $\Delta\rho_{core}$, $\Delta\rho_{methyl}$, $\Delta\rho_{MSP}$, excess electron density of lipid head group, core, methyl, and MSP, respectively; $X_{Roughness}$, interfacial roughness.

Table S1: Fitting results for MSP1E3D1:DMPC nanodisc data presented in Figure 1.

	**55 min**	**56 min**	**57 min**	**58 min**	**59 min**	**60 min**
N lipids	344 ± 45	334 ± 34	311 ± 43	309 ± 37	300 ± 50	293 ± 83
A (Å^2)	45.8 ± 2.8	46.0 ± 1.9	46.0 ± 1.4	46.1 ± 1.3	46.0 ± 1.8	46.2 ± 3.5
Axis ratio	1.49 ± 0.33	1.42 ± 0.21	1.39 ± 0.18	1.43 ± 0.15	1.43 ± 0.22	1.53 ± 0.38
$X_{Roughness}$	2.7 ± 2.0	2.7 ± 1.3	2.6 ± 0.9	2.6 ± 1.2	2.7 ± 2.2	2.7 ± 1.3
R_1 (Å)	61.2	59.0	56.3	57.0	56.2	57.3
R_2 (Å)	35.4	41.0	41.4	40.4	39.8	37.5
$H_{bilayer}$ (Å)	46.5	46.4	46.4	46.4	46.4	46.3
H_{core} (Å)	32.2	32.2	32.2	32.2	32.2	32.2
H_{methyl} (Å)	4.8	4.8	4.8	4.8	4.8	4.8
W_{belt} (Å)	8.0	8.2	8.6	8.6	8.7	8.7
$\Delta\rho_{head}$ (e/Å^3)	0.168	0.168	0.167	0.166	0.167	0.166
$\Delta\rho_{core}$ (e/Å^3)	-0.029	-0.029	-0.030	-0.030	-0.030	-0.030
$\Delta\rho_{methyl}$ (e/Å^3)	-0.171	-0.171	-0.171	-0.172	-0.171	-0.172
$\Delta\rho_{MSP}$ (e/Å^3)	0.150	0.143	0.137	0.138	0.137	0.139

Table S2 Fitting results from the MSP1E3D1:DMPC data presented in Figure 2. R_g and D_{max} are determined from the experimental data using ATSAS.

	10C (gel phase)	30C (liquid phase)
Nlipids	327 ± 15	327 (fixed)
A (Å2)	46.1 ± 1.0	52.6 ± 1.1
Axis ratio	1.48 ± 0.10	1.36 ± 0.08
$X_{Roughness}$	2.7 ± 0.6	2.9 ± 0.6
R1 (Å)	57.2	58.6
R2 (Å)	39.5	43.9
$H_{bilayer}$ (Å)	46.3	42.3
H_{core} (Å)	32.1	29.3
H_{methyl} (Å)	4.8	4.4
W_{belt} (Å)	8.6	8.1
$\Delta\rho_{head}$ (e/Å^3)	0.166	0.147
$\Delta\rho_{core}$ (e/Å^3)	-0.030	-0.041
$\Delta\rho_{methyl}$ (e/Å^3)	-0.171	-0.178
$\Delta\rho_{MSP}$ (e/Å^3)	0.139	0.140
R_g (Å)	55.4 ± 0.3	62.1 ± 0.4
D_{max} (Å)	139	150

Table S3 Fitting results for the nanodisc data presented in Figure 3, for the four different nanodisc constructs.

	MSP1D1:DMPC	MSP1D1:DPPC	MSP1E3D1:DMPC	MSP1E3D1:DPPC
N lipids	188 ± 19	188 ± 19	322 ± 17	348 ± 21
A (Å^2)	49.4 ± 2.4	47.3 ± 1.7	48.9 ± 0.9	46.2 ± 0.7
Axis ratio	1.19 ± 0.18	1.19 ± 0.22	1.38 ± 0.09	1.27 ± 0.13
$X_{Roughness}$	2.7 ± 1.3	2.9 ± 0.9	2.7 ± 0.5	2.9 ± 0.5
R_1 (Å)	41.9	41.0	58.8	57.1
R_2 (Å)	35.3	34.5	42.6	44.9
$H_{bilayer}$ (Å)	44.3	49.6	44.7	50.5
H_{core} (Å)	30.7	35.7	31.0	36.4
H_{methyl} (Å)	4.6	4.3	4.6	4.4
W_{belt} (Å)	7.8	8.3	8.3	8.6
$\Delta\rho_{head}$ (e/Å^3)	0.155	0.167	0.155	0.169
$\Delta\rho_{core}$ (e/Å^3)	-0.037	-0.033	-0.037	-0.032
$\Delta\rho_{methyl}$ (e/Å^3)	-0.175	-0.158	-0.175	-0.158
$\Delta\rho_{MSP}$ (e/Å^3)	0.137	0.118	0.133	0.116

Table S4 Input parameters used to generate the simulated scattering intensity presented in Figure 5, based on the method described in the text.

	Fluid bilayer structure with multiple roughness values	WillItFit bilayer structure with a single roughness value
$X_{Roughness}$	2.4 (head)<br>3.5 (core)<br>4.8 (methyl)<br>3.7 (bilayer in-plane)<br>3.7 (protein belt)	3.7
R1 (Å)	58.6	57.9
R2 (Å)	43.9	43.8
$H_{bilayer}$ (Å)	41.5	39.9
H_{core} (Å)	26.8	27.7
H_{methyl} (Å)	6.4	4.1
W_{belt} (Å)	8.1	8.6
$\Delta\rho_{head}$ (e/Å^3)	0.136	0.157
$\Delta\rho_{core}$ (e/Å^3)	-0.032	-0.035
$\Delta\rho_{methyl}$ (e/Å^3)	-0.158	-0.174
$\Delta\rho_{MSP}$ (e/Å^3)	0.160	0.122

3. Form factor of an irregular disc shape

Figure S3 An comparison between the form factors calculated from an irregular disc shape and an elliptical shape using FFT. The dashed elliptical outlines in the two density maps are identical and provide a size comparison.

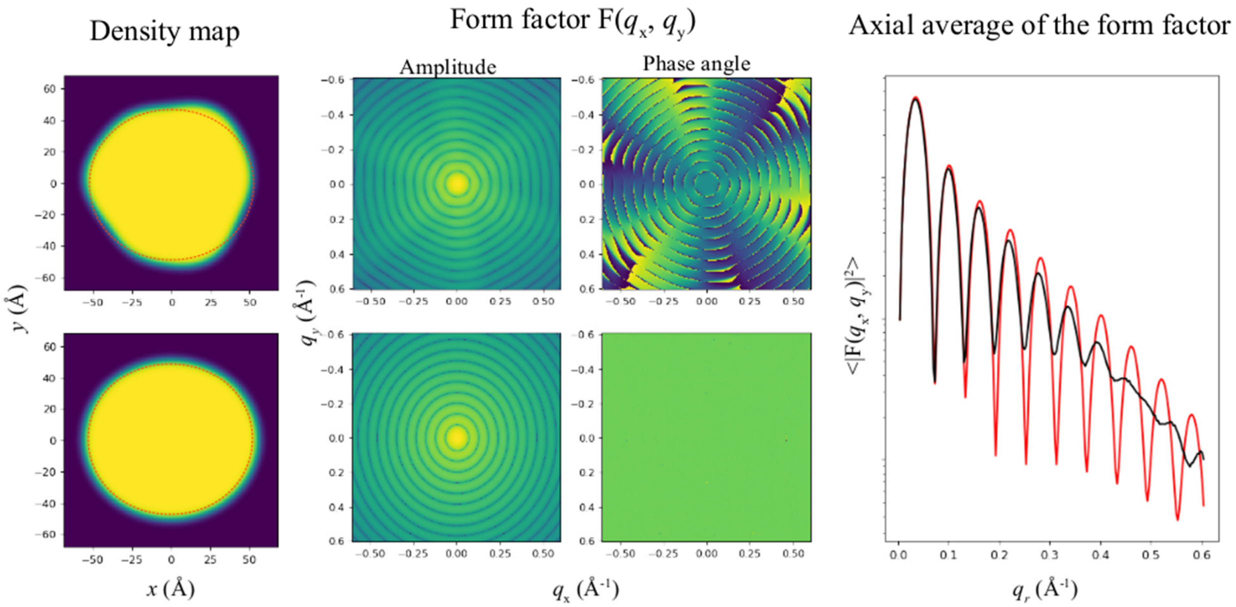

Supplement: Supplementary file 1 [file j-51-00157-sup1.pdf]
